# Tenth Annual Report of the Board of Education, of the City of Chicago

**Published:** 1864-05

**Authors:** 


TENTH ANNUAL REPORT OF THE BOARD OF EDUCATION, OF THE CITY OF CHICAGO; for the year ending Dec. 31st, 1863.

This is a pamphlet of 103 pages, containing the reports of the President of the Board of Education; the Superintendent of Public Instruction; the Principal of the Chicago High School; the Rules of the Board of Education; the list of Schools, Principals, and Assistants, with their Salaries; and a variety of other facts of interest to those engaged in the business of education.

The reports are well written, and give a very full account of the present condition of our liberal and enlightened system of Public Schools.

